# Efficacy of leflunomide combined with ligustrazine in the treatment of rheumatoid arthritis: prediction with network pharmacology and validation in a clinical trial

**DOI:** 10.1186/s13020-019-0247-8

**Published:** 2019-08-02

**Authors:** Chi Zhang, Daogang Guan, Miao Jiang, Chao Liang, Li Li, Ning Zhao, Qinglin Zha, Wandong Zhang, Cheng Lu, Ge Zhang, Jian Liu, Aiping Lu

**Affiliations:** 1 Institute of Basic Research in Clinical Medicine, China Academy of Chinese Medical Sciences, Nanxiaojie, Beijing, China; 2 School of Chinese Medicine, Hong Kong Baptist University, Kowloon Tong, Kowloon, Hong Kong; 3 School of Computer, Jiangxi University of Traditional Chinese Medicine, Nanchang, China; 4 Division of Rheumatology, The First Affiliated Hospital of Anhui University of Chinese Medicine, Anhui, China

**Keywords:** Rheumatoid arthritis, Leflunomide, Ligustrazine

## Abstract

**Background:**

Leflunomide (LEF) is a first-line disease-modifying antirheumatic drug (DMARD) for rheumatoid arthritis (RA). However, there are still a few nonresponders. It is logical to suggest that employing combinations including LEF that produce synergistic effects in terms of pharmacological activity is a promising strategy to improve clinical outcomes.

**Methods:**

We propose a novel approach for predicting LEF combinations through investigating the potential effects of drug targets on the disease signaling network. We first constructed an RA signaling network with disease-associated driver genes. Thousands of available FDA-approved and investigational compounds were then selected based on a drug-RA network, which was generated using an algorithm model named synergistic score that combines chemical structure, functional prediction and target pathway. We then validated our predicted combination in a prospective clinical trial.

**Results:**

Ligustrazine (LIG), a key component of the Chinese herb Chuanxiong and an approved drug in China, ranked first according to synergistic score. In the clinical trial, after 48 weeks, the American College of Rheumatology (ACR) 20 response rate was significantly lower (*P *< 0.05) in the LEF group [58.8% (45.4%, 72.3%)] than in the LEF + LIG group [78.7% (68.5%, 89.0%)]. Consistently, the erosion score was lower in patients treated with LEF + LIG than in those treated with LEF (0.34 ± 0.20 vs 1.12 ± 0.30, *P *< 0.05).

**Conclusions:**

Our algorithm combines structure and target pathways into one model that predicted that the combination of LEF and LIG can reduce joint inflammation and attenuate bone erosion in RA patients. To our knowledge, this study is the first to apply this paradigm to evaluate drug combination hypotheses.

**Electronic supplementary material:**

The online version of this article (10.1186/s13020-019-0247-8) contains supplementary material, which is available to authorized users.

## Background

Rheumatoid arthritis (RA) is a chronic inflammatory disease that, if left untreated, leads to functional disability, reduced health-related quality of life and premature mortality [1]. Different classes of immunomodulatory agents with distinct mechanisms of action are approved for RA treatment [2]. However, the current RA medications are only somewhat effective; they can be associated with side effects and potential toxicities [3], and there is ongoing debate regarding the effect of certain agents on the progression of bone erosion [4, 5]. While one strategy to improve RA therapy is to develop novel agents that may have greater efficacy, it is important to identify existing or novel classes of drugs that may complement one another in combination to provide synergistic benefit.

Leflunomide (LEF) is an isoxazol derivative used as a disease-modifying antirheumatic drug (DMARD) in the treatment of RA [6, 7]. It is structurally distinct from other DMARDs. LEF is one potential drug that could effectively replace MTX in the treatment of RA if intolerance to MTX or therapeutic failure occurs, and it is the first choice if MTX is contraindicated according to the European League Against Rheumatism (EULAR) recommendations for the treatment of RA [2]. Key findings of a systematic review suggest that LEF monotherapy has only partial superiority over methotrexate (MTX) in the population of patients who achieve an ACR clinical response [8]. A study with an observation period of 2.5 years reported less pronounced radiographic progression in patients treated with LEF than in those treated with MTX [9]. These results suggest that there are still a few nonresponders to LEF monotherapy and that there is a considerable unmet need for LEF combination therapy to supplement traditional DMARD therapy. To address this need, the investigators have seen growing enthusiasm for the development of LEF combinations for RA therapy [10].

Drug combinations have been widely used to treat complex diseases such as RA, cancer and infectious diseases [11]. A cornerstone for optimizing RA treatment strategies has been combination therapy with DMARDs [12, 13]. This strategy relies on the experience of oncologists to combine drugs with different mechanisms of action to achieve additive or synergistic effects without increasing toxicity. Although LEF combination therapy shows some promising results, most currently used LEF combination therapies were found in empirical ways [11], which limits the speed of discovery for new and more effective combinations. Thus, it is logical to use a systems pharmacology approach to find new combinations; if a LEF combination is able to fully cover the RA pharmacological network, or at least provide high coverage, then combination therapy with LEF and one complementary agent will be relatively more effective than LEF alone in producing significant treatment-related changes [14]. Network-based approaches can more explicitly indicate a possible mechanism of action and consequently specify a measure for predicting efficacy. Many studies have used various combinations of data mining methods to measure the efficiency of drug combinations [15–18]; Li et al. used the concepts of network centrality and disease similarity to prioritize drug combinations [19], Gottlieb and associates used the new method INferring Drug Interactions for predictions [20], and others have used the concept of synthetic lethality and available gene interaction data [21]. Despite the countless attempts, there are still many challenges, especially clinical uncertainties about the prediction.

Here, we propose an approach to evaluate the synergistic scores of combinations that applies a recommendation technique based on HitPick, Similarity Ensemble Approach, STITCH, and Swiss Target Prediction. This technique, combined with the constructed disease signaling network and predicted drug targets, was used to identify LEF combinations for RA treatment, and we also provide clinical validation from a prospective trial in which the predicted LEF combination was used for RA treatment.

## Materials and methods

We reported this study according to the Minimum Standards of Reporting Checklist.

### Prediction of a drug that will synergize with LEF for RA treatment

#### Methods

We defined an RA disease signaling network by integrating gene expression data from the publicly available datasets MalaCards, DisGeNET and EDGAR. To obtain the targets of LEF and marketed drugs, commonly used software, i.e., HitPick [22], Similarity Ensemble Approach (SEA) [23], STITCH [24], and Swiss Target Prediction [25], were employed. All chemical structures were prepared and converted into canonical SMILES using Open Babel Toolkit (version 2.4.1). Protein–protein interaction (PPI) data were derived from the public databases BioGRID, STRING, Dip, HPRD, Intact, Mint and Reactome. Cytoscape 3.5.1 [26], an open-source software platform for visualizing complex networks, was employed to visualize the networks.

Synergistic score of the target network: The constructed disease signaling network and predicted drug targets were used to prioritize drug combinations by combining the following synergistic scores. Given two candidate drugs, di and dj, suppose ${\text{d}}_{t} \in C_{k}$ and ${\text{d}}_{j} \in C_{n}$; ${\text{T}}_{k} = \{ t_{k1} ,t_{k2} , \ldots ,t_{km} \}$ denotes the targets of di in C_k_, and ${\text{T}}_{h} = \{ {\text{t}}_{{{\text{h}}1}} ,{\text{t}}_{{{\text{h}}2}} , \ldots ,{\text{t}}_{\text{hn}} \}$ denotes the targets of dj in Ch. In the reconstructed disease signaling network, two drug synergistic scores are defined as follows.

$$ S_{tnetwork} = \frac{{\sum\nolimits_{i} {CS(t_{ki} )\exp \left( {\frac{{D(t_{ki} ,T_{h} )}}{{n^{2} }}} \right)} }}{{\sum\nolimits_{i} {CS{t_{ki} }} }} + \frac{{\sum\nolimits_{j} {CS(t_{hj} )\exp \left( {\frac{{D{t_{hj} ,T_{k} }}}{{m^{2} }}} \right)} }}{{\sum\nolimits_{j} {CS(t_{hj} )} }}, $$

where $CS(t_{ki} )$ is the centrality score of target *t*_*ki*_ in the reconstructed disease signaling network, and it is the sum of the betweenness (Bn), closeness (Cn) and PageRank (Pr) scores of protein *t*_*ki*_: ${\text{CS}}({\text{t}}_{ki} ) = Bn({\text{t}}_{ki} ) + Cn({\text{t}}_{ki} ) + \Pr ({\text{t}}_{ki} ).$

These are three different but correlated centrality measurements, and the reason for combining them is to obtain a robust centrality score. The min of D {t_ki_, Th} is the minimum shortest path from tki to Th. The target network synergistic score, S1 (i, j), prefers drug combinations whose targets are in the center (hubs) of the disease signaling network and are closely connected.

Synergistic function score: The synergistic function score is defined as:

$$ S_{function} = \frac{{\sum\nolimits_{i,j} {\frac{{2\log_{2} \text{max} {p(A)}}}{{(\log_{2} p(GO_{ki} ) + \log_{2} p(GO_{hj} ))}}} }}{(m + n)(m + n - 1)} $$

where Sim(tki, thj) is the semantic similarity of the gene ontology (GO) annotations of tki and thj [27, 28], which is computed based on the overlap of GO terms associated with tki and thj; GOki is the GO term associated with tki, A is a GO term that is an ancestor of both GOki and GOhj, $p(GO_{ki} ) = Freq(GO_{ki} )/Max(Freq)$, and Freq(GOki) is the frequency of the GO term GOki in GO annotations taken from the GO database. Max (Freq) is the maximum occurrence frequency of GO terms associated with all the targets and predicted drug targets among the GO annotations.

Synergistic score of 2D similarity: LINGO refers to q-character substrings of a SMILES text [29]. LINGO representation of compounds has been used as input for Quantitative Structure–Property Relationships (QSPR) models and for the calculation of intermolecular similarities. A SMILES string of length n can be represented with (n − (q − 1)) q-length substrings (LINGOs). The original method requires SMILES strings to be canonical, and the LINGO length is fixed as q = 4. Before the LINGO creation process, all ring numbers in the SMILES string are set to ‘0’. Then, the LINGOsim function is used to calculate the similarity between two SMILES strings *d*_*i*_ and *d*_*j*_ with the Tanimoto coefficient based on their LINGO profiles.

$$ S_{2dsimilarity} = \frac{{\sum\nolimits_{k = 1}^{m} {1\frac{{N_{{d_{i} ,k}} - N_{{d_{j} ,k}} }}{{N_{{d_{i} ,k}} + N_{{d_{j} ,k}} }}} }}{m}, $$

where m is the total number of unique LINGOs created from *d*_*i*_ and *d*_*j*_, $N_{{d_{i} ,k}}$ represents the frequency of LINGOs of type k in compound *d*_*i*_, and $N_{{d_{j} ,k}}$ represents the frequency of LINGOs of type k in compound *d*_*j*_.

Synergistic score of 3D similarity: We performed pharmacophoric calculations using the Schrödinger package in Phase and assessed the 3D similarity of all pairs of drugs. The most stable previously determined 3D structure of each drug was used as a template. Shape screening generated different conformers for the rest of the drugs and aligned them each to a template to identify common pharmacophoric features in each pair of drugs. The calculation yielded a 3D similarity score called the Phase Sim property that measured the overlapping volume between the same types of pharmacophoric features in each pair of superimposed drugs [30]. The 3D score spans values between 0 (minimum 3D similarity) and 1 (maximum 3D similarity), and it is defined as

$$ S_{2Dsimilarity} = \frac{{O(d_{i} ,d_{j} )}}{{\text{max} (O(d_{i} ,d_{i} ),O(d_{j} ,d_{j} ))}}, $$

where $O(d_{i} ,d_{j} )$ is the overlap of pharmacophoric sites between drugs *d*_*i*_ and *d*_*j*_, and $\text{max} (O(d_{i} ,d_{i} ),O(d_{j} ,d_{j} ))$ is the maximum of the self-overlaps. The total synergistic similarity score is $S_{similarity} = S_{2dsimilarity} + S_{3Dsimilarity} .$

### Results

#### Construction of the RA network

**Fig. 1:**
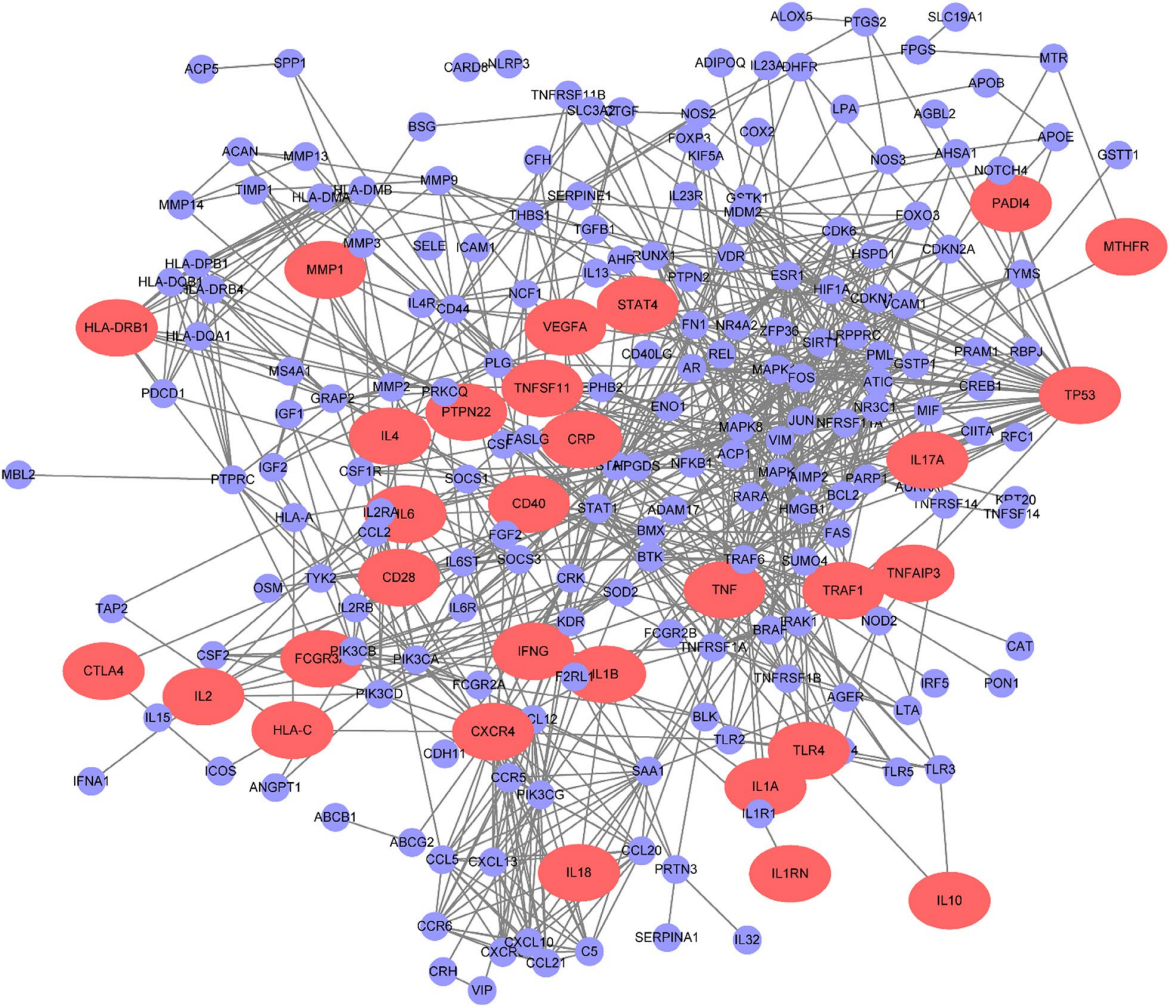
High confirmed protein–protein interaction network of rheumatoid arthritis (RA). Red nodes represent more than 30 published evidence from publicly available dataset MalaCards, DisGeNET and eDGAR

To construct the RA network, 277 RA-associated genes were identified from the MalaCards, DisGeNET, PsyGeNET, OMIM, and DISEASES public databases and the literature. It is known that genes and their encoded proteins function in concert rather than in an isolated manner. In this study, a dataset of human protein–protein interactions derived from the public databases BioGRID, STRING, Dip, HPRD, Intact, Mint and Reactome, including 18,740 proteins (nodes) and 430,399 interactions (edges), was used as a background network. Then 277 RA associated genes were mapped to the background network and removed the nodes without any edges to construct the potential RA network, which represented a group of interacting proteins playing critical roles in the pathogenesis of RA (Fig. 1).

#### Construction of the LEF-target network

**Fig. 2:**
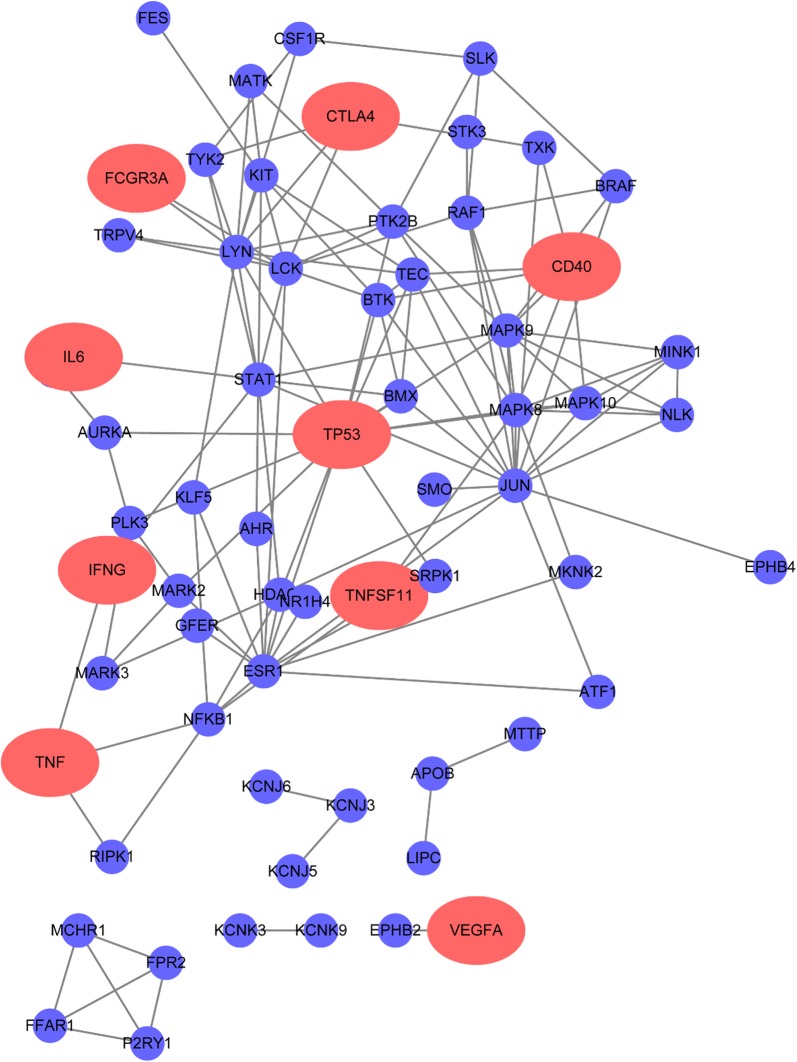
Predicted targeting protein–protein interaction network of Leflunomide. Red nodes represent proteins overlap with the high confidence proteins which have more than 30 published evidence from publicly available datasets in RA protein–protein interaction network

In total, 146 target genes were identified. Then, these the target genes were mapped to the constructed PPI background network and removed the nodes without any edges to construct the potential LEF-response network, which represented a group of interacting proteins playing critical roles in the response of RA (Fig. 2).

#### Construction of bioinformatic model to screen drug synergize with LEF for RA treatment

As mentioned above, the target network LEF is not enough to cover the RA-related Pathogenesis Network Confirmed by published databases. New methods need to be developed to detect the combination of a drug with LEF that can cover RA-related Pathogenesis Network at the greatest extent level, thus we design an approach for predicting a drug to synergize with LEF for RA treatment. In addition to the target network, we also consider the structural similarity of compounds and the functional similarity of target proteins (Additional file 1: Figure S1).

#### Ligustrazine predicted as the optimal drug combined with LEF

**Fig. 3:**
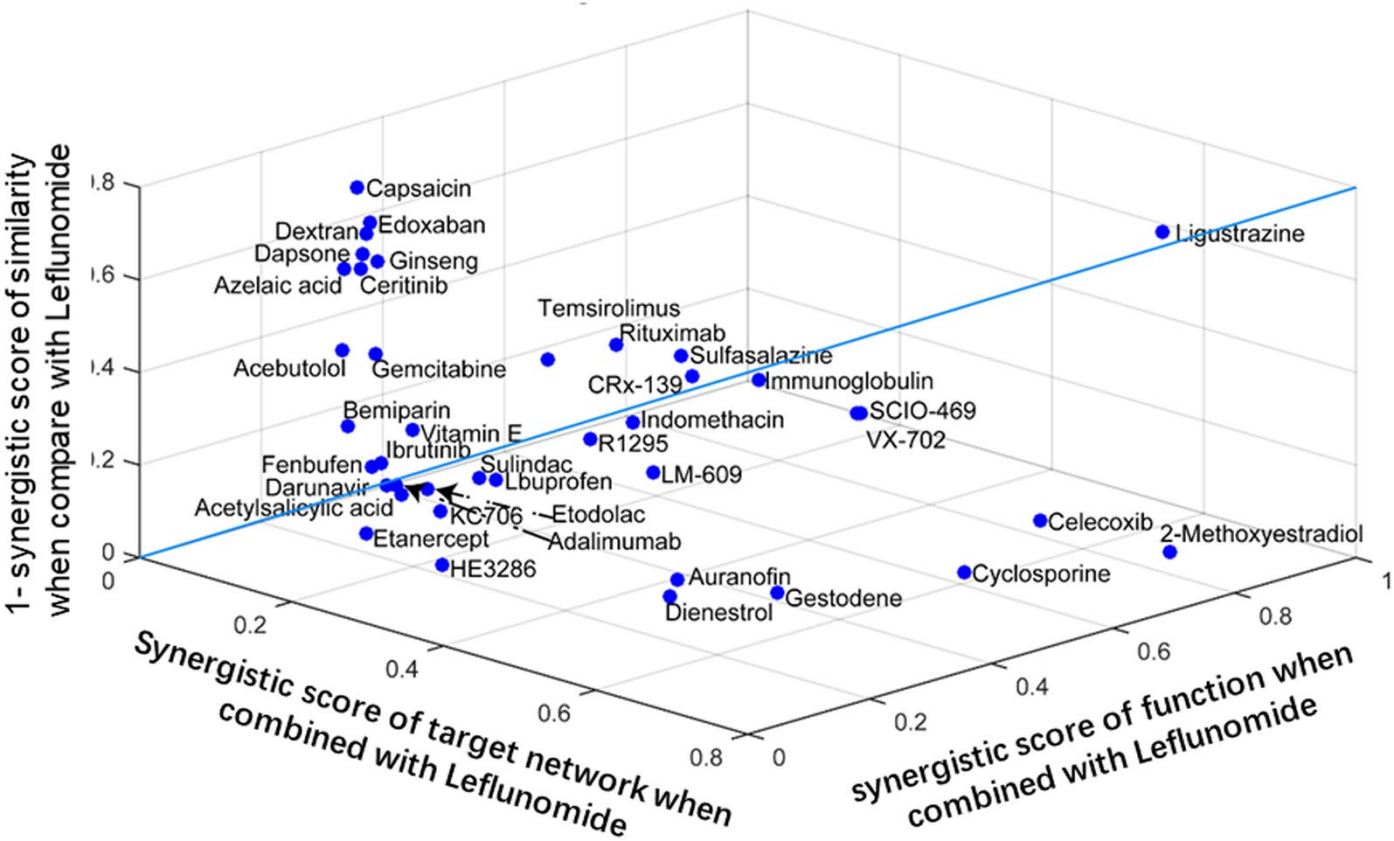
Ligustrazine predicted as the optimal drug combined with Leflunomide by the 3D plot of scores of chemical similarities, target network and function analysis. Blue line is diagonal of 3-dimensional graph

To identify a candidate drug that could cover the RA network in combination with LEF, we performed the above model to screen marketed drugs that could be combined with LEF to obtain high synergistic scores, including synergistic scores of target network, function and structural similarity. Synergistic scores were calculated for FDA-approved drugs and 500 comprehensive natural products from herbs using their target networks, function analysis and structural similarity. The virtual screening data demonstrated that ligustrazine (LIG) had higher scores at target network, function and structure similarity, respectively, when compared to LEF alone. After further comprehensive analysis, LIG near the other end of the diagonal, away from the origin with the highest synergistic score was identified as the optimal one (Fig. 3). LIG is a structural compound derived from Chinese herbs that has been approved by National Medical Products Administration (NMPA) for clinical application.

### Validation of the predicted antirheumatic drug combination for RA in an RCT

LIG was approved to treat coronary heart disease in China. Interestingly, LIG was proposed as adjunct therapy for active RA in an academic hospital affiliated with Anhui University of Chinese Medicine, Anhui, China. Therefore, it is ethical to validate the efficacy of the predicted drug combination in a prospective clinical trial.

#### Trial design

This two-arm RCT was conducted from November 2014 to November 2017 in the First Affiliated Hospital of Anhui University of Traditional Chinese Medicine (FAH-AUTCM). The trial was registered at the Chinese Clinical Trial Registry of Clinical Trials (http://www.chictr.org.cn/index.aspx) with the ID ChiCTR-TRC-10001014.

#### Sample size

A sample size of 60 participants per group was needed to provide 80% power to detect a 26% improvement in the ACR20 in the combination group compared with the LEF alone group, assuming an ACR20 of approximately 50% for the LEF alone group and 10% drop-out.

#### Participants

One hundred twenty-three RA patients diagnosed by the 1987 American Rheumatism Association criteria and the 2010 American College of Rheumatology (ACR)/European League against Rheumatism (EULAR) criteria were enrolled in 2014–2017 at FAH-AUTCM.

#### Selection criteria

The inclusion criteria were as follows: (1) meet the 1987 American Rheumatism Association criteria and 2010 ACR/EULAR criteria; (2) ≥ 18 years of age; and (3) have active disease. The exclusion criteria were as follows: (1) known cardiovascular, lung, or liver disease; (2) use of oral corticosteroids (10 mg/kg or less prednisone equivalent) or nonsteroidal anti-inflammatory drugs (NSAIDs) and must have been on a stable dose for at least 4 weeks before screening; (3) platelets < 100*10^9^/L; (4) pregnancy; (5) breastfeeding; (6) and use of lipid lowering agents.

#### Randomization and blinding

A total of 123 participants were randomized to the combination therapy group or the LEF alone group by the central randomization system provided by the China Academy of Chinese Medical Sciences, which adopted computer telephone integration (CTI) technology to integrate computers, internet and telecom. The random number list was assigned by interactive voice response (IVR) and interactive web response (IWR). The independent drug administrators received group information based on a random number, and then, they assigned the study drug to the nurses. Data analysis was performed by a statistician who was blinded to patient allocation.

#### Interventions and data collection

All data were collected using a checklist to record the observational results. The participants were randomly divided into two groups: (1) the intervention group received LIG (injection, 0.12 g, solubilized in 5% GS/NS, 100 ml daily for 6 days per week for 2 weeks per month) and LEF (20 mg, qd, po) (62 subjects), and (2) the control group received LEF without LIG (61 subjects). Ligustrazine hydrochloride injection (NMPA Approval number: H20050593) was from Jiangsu Pingguang Pharmaceutical Co., Ltd., China. All patients were evaluated at weeks zero, twelve, twenty-four, and forty-eight by two rheumatologists (disagreements were resolved by consensus or, when necessary, by a third rheumatologist). Non-steroidal anti-inflammatory drugs (NSAIDs) were actively discouraged and parenterally administered corticosteroids were permitted as clinically indicated. Each patient’s response to treatment was evaluated using the ACR20 as the primary outcome at week 48. ACR 20 has a positive outcome if 20% improvement in tender or swollen joint counts were achieved as well as a 20% improvement in at least three of the other five criteria. During each visit, secondary endpoints were measured in both study groups, including erosion score [31], C-reactive protein (CRP) and erythrocyte sedimentation rate (ESR). Safety parameters evaluated in this study included general and systemic clinical examinations, laboratory investigations and assessments of all adverse events.

#### Statistical methods

All the statistical data in this project were analyzed by a contract service from Bioinformedicine (San Diego, CA, USA, http://www.bioinformedicine.com/index.php). The *t* test, Chi square test, and ANOVA were used for data analysis. *P*-values less than 0.05 were considered statistically significant.

#### Results

**Fig. 4:**
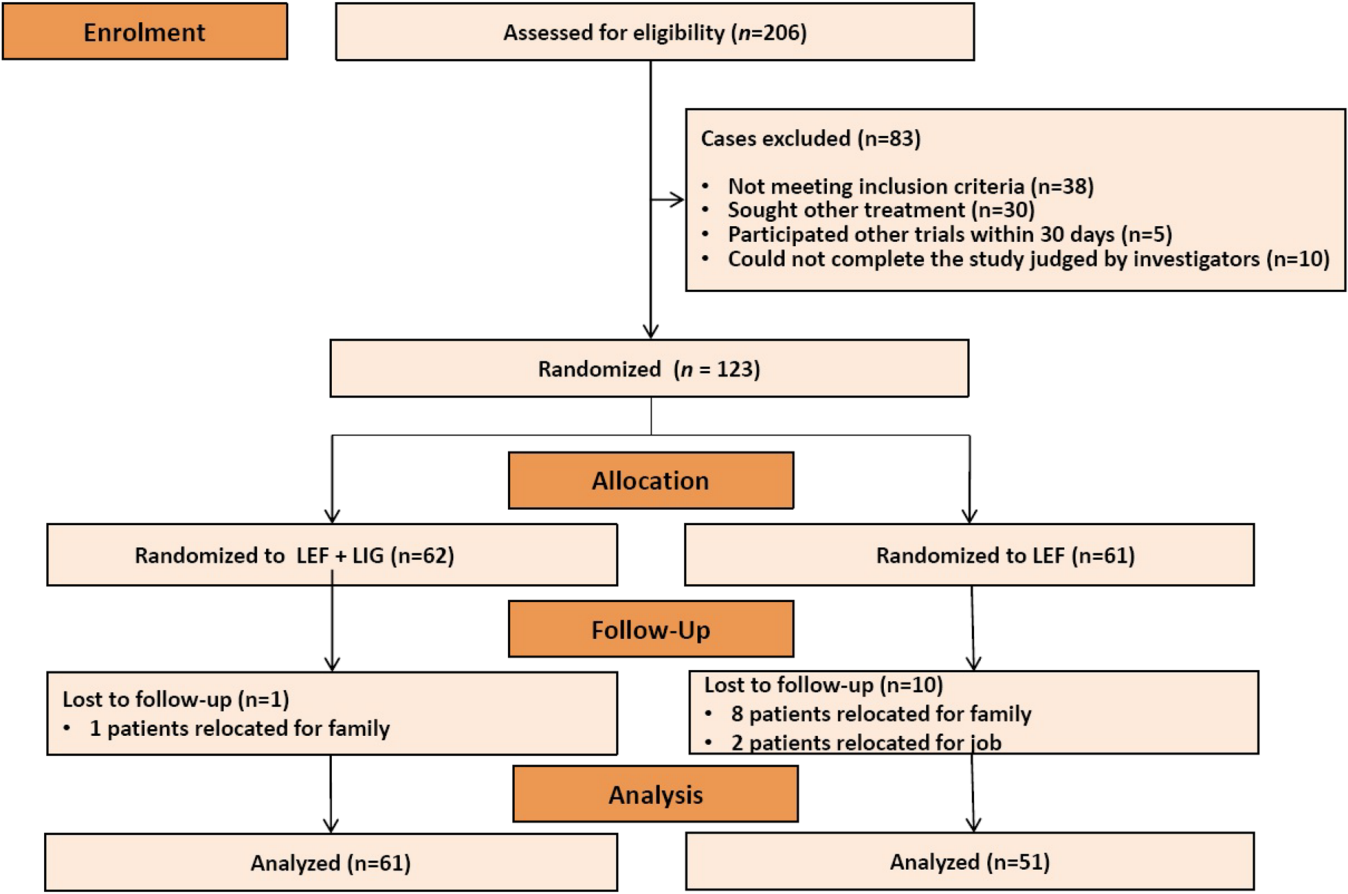
Trial (combination of leflunomide and ligustrazine in treatment of rheumatoid arthritis) flow chart

**Fig. 5:**
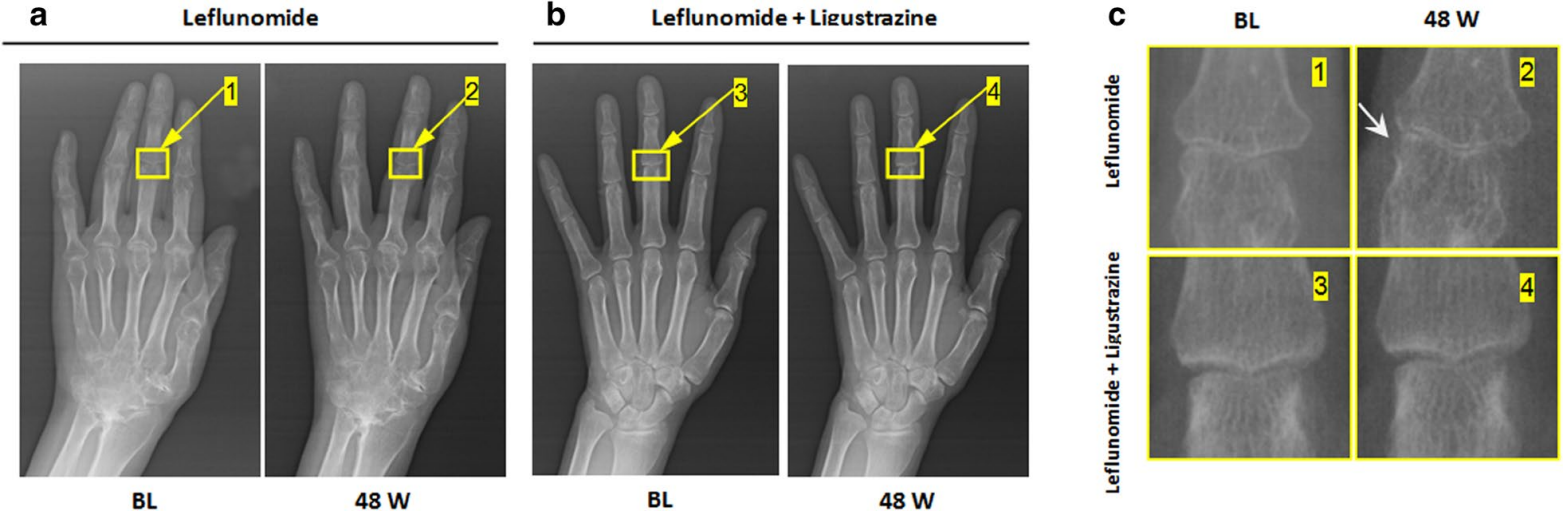
The representative hand X-ray radiographs (left) and enlarged images (right) showing bone cortex erosion (indicated by arrows) at the interphalangeal joint in rheumatoid arthritis (RA) patients before (baseline, BL) and after treatment with either leflunomide (LEF, n = 51) or a combination of leflunomide and ligustrazine (LEF + LIG, n = 61) at week 48, respectively

A total of 123 patients were recruited and randomly assigned into the LEF group or the LEF and LIG combination (LEF + LIG) group. During the trial, 8 patients in the LEF group and 1 patient in the LEF + LIG group moved to other cities with their families, and 2 patients in the LEF group were sent by their companies to work in other cities; these patients were lost to follow-up and discontinued the study. In total, 51 patients in the LEF group and 61 patients in the LEF + LIG group completed the study. Demographic data showed that 76.5% and 75.4% of the patients were female in the LEF and LEF + LIG groups, respectively. Figure 4 shows the CONSORT flow diagram of the trial. The mean age of the patients was 52.4 ± 10.9 years in the LEF group and 54.1 ± 11.0 years in the LEF + LIG group. There were no significant differences between groups in the mean age of patients (*P *= 0.84) or any demographic parameter (Additional file 2: Table S1). According to the clinical findings, the ACR20 response rate was significantly lower (*P *= 0.02) in the LEF group [58.8% (45.4%, 72.3%)] than in the LEF + LIG group [78.7% (68.5%, 89.0%)]. The between-group difference was − 19.9% (95% CI − 36.8%, − 2.9%). Based on X-ray radiographs, the change in the erosion score was 0.34 ± 0.20 in the LEF + LIG group and 1.12 ± 0.30 in the LEF group (*P *< 0.05). There was an obvious increase in the erosion score from baseline in the LEF group after treatment. Representative hand X-ray radiographs are presented in Fig. 5. Significantly more patients in the LEF + LIG group than in the LEF group achieved a 20% improvement in CRP (86.96% vs. 57.14%) and ESR (80.00% vs. 36.36). There were no persistent changes from baseline in laboratory parameters in any group; these results are presented in Additional file 3: Table S2. A total of 28 adverse events (AEs) were reported in 17 subjects among treatment groups, and no serious AE was reported during the study. The distribution of AEs was comparable between two groups. There were 12 AEs reported in seven subjects in the test group, whereas in the reference group 16 AEs were reported by ten subjects. Erythra and headache were commonly reported in both groups.

## Discussion

In this paper, we present a synergistic score evaluation, a computational method for characterizing drug interactions. There is a conceptual difference between the synergistic score and many other concepts related to drug combinations. Unlike existing integrative analyses that treat structure and target pathways as two separate processes, our approach combines these two types of data into a single model, which is more biologically meaningful. One challenge is that the combination kinetic function is essentially nonlinear, which makes it difficult to develop computational methodologies [32, 33]. Here, we utilized Taylor expansion to convert the nonlinear kinetic function to a polynomial function, which provides a general mathematic form to simultaneously involve different combinations. By assuming that each combination has a probability of being involved in a potential function, we are able to construct the model equation. Solving the model equations can lead to the determination of key combinations.

Furthermore, the candidate LEF combination showed good correlation with the clinical trial results. Despite large investments in drug combinations, the overall success rate of combination therapies during clinical development remains low. The main reason for these failures is the lack of efficacy in clinical trials. This trial revealed a significant improvement in the ACR20 response and changes in the erosion score. According to the obtained results, simultaneous treatment with LIG and LEF led to a significant reduction in CRP. Interestingly, LIG was recently shown to improve the ACR response. A clinical trial in active RA evaluating the efficacy of LIG plus MTX, hydroxychloroquine (HCQ) and loxoprofen [34] has shown consistent results, but the sample size was small, and the trial was not registered. After our trial, LIG may be used as an adjunct along with LEF, which is routinely recommended in practice.

There are some limitations of our method that can be improved and other challenges for further investigation. First, a limitation of the current prediction method is that the constructed RA signaling network may not be entirely accurate. Other methods should be explored. To further improve the prediction, other knowledge can be integrated. Second, it will be important to experimentally confirm potentially synergistic mechanisms to assess the impact of local pathways and subnetworks in the overall RA signaling network. There are unanswered questions and issues worthy of further exploration, such as show improved RNAs or proteins as marker in patients or in cultured cells after treatment of combination of LEF and LIG compared to LEF alone. Furthermore, this study applies to the small molecule drug LEF, and it will be important to further validate other drugs.

## Conclusions

Our present research provides a new direction for the treatment of RA with combination therapy, with the hope that this strategy could be clinically exploited in the future. This study also provides a strategy for discovering drug combination-based precision medicine for cases of specific drug treatment failure. A candidate marketed drug could be identified to have potential efficacy in combination to address the above mentioned failure. The strategy in this study will be illuminating for addressing other treatment failures in various diseases.

## Additional files

**Additional file 1: Figure S1.** Diagram of combined drug screening model. A. Target network combination score. B. Function analysis combination score. C. Structure similarity combination score. D. The mathematical model. E. The output results. Red nodes mean high confident evidence from published reports. Blue line represents the diagonal of the 3D graph.

**Additional file 2: Table S1.** The characteristics for 112 patients with rheumatoid arthritis.

**Additional file 3: Table S2.** Biochemistry and hematology parameters for 112 RA patients at week 0, 24, 48.

## Data Availability

The materials and data of this study are available from the corresponding author on reasonable request.

## Abbreviations

LEF: leflunomide
DMARDs: disease-modifying antirheumatic drugs
RA: rheumatoid arthritis
LIG: ligustrazine
ACR: American College of Rheumatology
SEA: similarity ensemble approach
GO: gene ontology
QSPR: Quantitative Structure–Property Relationships
CFDA: China Food and Drug Administration
EULAR: European League against Rheumatism
CTI: computer telephone integration
IVR: interactive voice response
IWR: interactive web response
CRP: C-reactive protein
ESR: erythrocyte sedimentation rate
HCQ: hydroxychloroquine
