# Supplementary figures and images for: Efficacy of leflunomide combined with ligustrazine in the treatment of rheumatoid arthritis: prediction with network pharmacology and validation in a clinical trial
Source: Chin Med. 2019 Aug 2;14:26. doi: 10.1186/s13020-019-0247-8 (PMC6679497; doi:10.1186/s13020-019-0247-8)

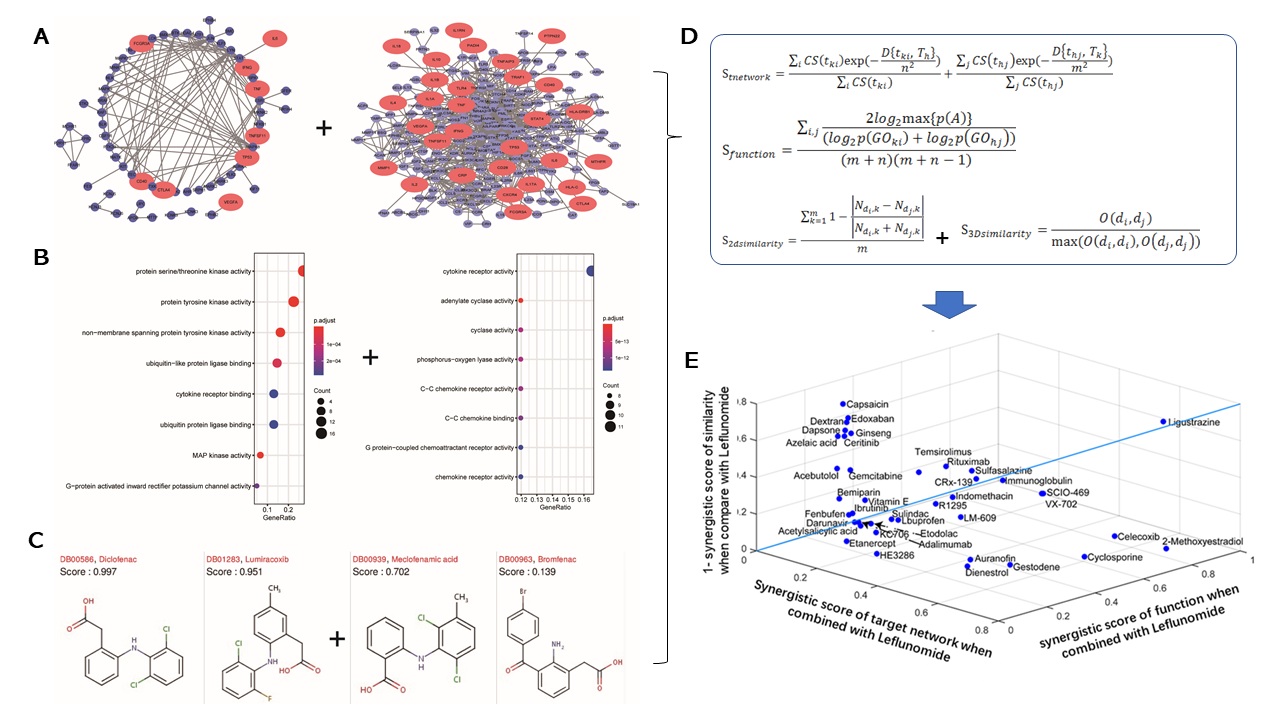

Supplement: Supplementary file 1 — Additional file 1: Figure S1. Diagram of combined drug screening model. A. Target network combination score. B. Function analysis combination score. C. Structure similarity combination score. D. The mathematical model. E. The output results. Red nodes mean high confident evidence from published reports. Blue line represents the diagonal of the 3D graph. [file 13020_2019_247_MOESM1_ESM.jpg]
