# Supplementary material for: Efficacy of leflunomide combined with ligustrazine in the treatment of rheumatoid arthritis: prediction with network pharmacology and validation in a clinical trial
Source: Chin Med. 2019 Aug 2;14:26. doi: 10.1186/s13020-019-0247-8 (PMC6679497; doi:10.1186/s13020-019-0247-8)
Supplement: Supplementary file 2 — Additional file 2: Table S1. The characteristics for 112 patients with rheumatoid arthritis. [file 13020_2019_247_MOESM2_ESM.docx]

**Table S1**

**The characteristics for 112 patients with rheumatoid arthritis**

| **Characteristics** | **LEF group (n=51)** | **LEF + LIG group (n=61)** |
| --- | --- | --- |
| **Male/Female** | 12/39 | 15/46 |
| **Age (years)** | 52.4 ± 10.9 | 54.1 ± 11.0 |
| **Duration of disease (months)** | 12.2 ± 6.1 | 11.7 ± 8.1 |
| **Rheumatoid factor positive n (%)** | 86% | 79% |
| **Number of tender joints** | 10.9 ± 12.1 | 11.12 ± 14.08 |
| **Number of swollen joints** | 11.1 ± 7.5 | 12.31 ± 8.61 |
| **Erosion Score** | 38.2 ± 4.2 | 38.8 ± 4.3 |
| **DMARD used before study (patients)** | 9 (18)^†^ | 8 (13)^†^ |

^†^Number of patients (percentage). Leflunomide=LEF; Ligustrazine=LIG; DMARD=disease-modifying antirheumatic drug
