# Supplementary material for: Efficacy of leflunomide combined with ligustrazine in the treatment of rheumatoid arthritis: prediction with network pharmacology and validation in a clinical trial
Source: Chin Med. 2019 Aug 2;14:26. doi: 10.1186/s13020-019-0247-8 (PMC6679497; doi:10.1186/s13020-019-0247-8)
Supplement: Supplementary file 3 — Additional file 3: Table S2. Biochemistry and hematology parameters for 112 RA patients at week 0, 24, 48. [file 13020_2019_247_MOESM3_ESM.docx]

**Table S2**

**Biochemistry and hematology parameters for 112 RA patients at week 0, 24, 48**

| Variables  (mg/dl) | LEF group (n=51) | | | LEF + LIG group(n=61) | | |
| --- | --- | --- | --- | --- | --- | --- |
|  | BL | 24 weeks | 48 weeks | BL | 24 weeks | 48 weeks |
| BUN | 13.21 ± 9.38 | 12.28 ± 10.03 | 13.09 ± 9.32 | 13.09 ± 8.76 | 14.99 ± 9.21 | 15.01 ± 8.86 |
| Uric acid | 5.06 ± 1.59 | 6.09 ± 1.78 | 6.12 ± 1.07 | 6.10 ± 0.76 | 6.11 ± 1.91 | 6.12 ± 1.21 |
| Cholesterol | 1.65 ± 2.32 | 1.23 ± 2.09 | 1.09 ± 2.32 | 1.09 ± 1.76 | 1.18 ± 1.12 | 1.23 ± 1.06 |
| Triglyceride | 168.62 ± 39.8 | 172.88 ± 89.2 | 182.22 ± 69.3 | 176.93 ± 86.1 | 182.90 ± 78.65 | 185.76 ± 90.09 |
| AST | 0.96 ± 3.86 | 1.05 ± 1.98 | 0.76 ± 5.98 | 1.06 ± 2.07 | 1.32 ± 3.42 | 1.25 ± 2.96 |
| ALT | 1.45 ± 2.52 | 1.18 ± 2.03 | 0.98 ± 1.87 | 1.65 ± 3.52 | 1.09 ± 1.08 | 1.65 ± 2.01 |

ALT, Alanine aminotransferase; AST, aspartate aminotransferase; BUN, blood urea nitrogen; BL: baseline.
